# Effect of Treatment and Nutrition on Glycemic Variability in Critically Ill Patients

**DOI:** 10.3390/ijerph19084717

**Published:** 2022-04-13

**Authors:** Cezary Kapłan, Alicja Kalemba, Monika Krok, Łukasz Krzych

**Affiliations:** 1 Students’ Scientific Society, Department of Anesthesiology and Intensive Care, School of Medicine in Katowice, Medical University of Silesia, 14 Medyków Street, 40-752 Katowice, Poland; cezary.kaplan@gmail.com (C.K.); mokakrok@gmail.com (M.K.); 2 Department of Anesthesiology and Intensive Care, School of Medicine in Katowice, Medical University of Silesia, 14 Medyków Street, 40-752 Katowice, Poland; l.krzych@wp.pl

**Keywords:** glycemic variability, glycemic control, diabetes, insulin therapy, treatment, complications, intensive care

## Abstract

Nondiabetic hyperglycemia is a dangerous metabolic phenomenon in the intensive care unit. Inattentive treatment of glycemic disorders is a serious health hazard promoting negative outcomes. The aim of our study was to assess glycemic variability and its basic determinants, and to verify its relationship with mortality in patients hospitalized in a mixed ICU (intensive care unit). The medical records of 37 patients hospitalized 13 January–29 February 2020 were analyzed prospectively. The BG (blood glucose) variability during the stay was assessed using two definitions, i.e., the value of standard deviation (SD) from all the measurements performed and the coefficient of variation (CV). A correlation between the BG variability and insulin dose was observed (SD: R = 0.559; *p* < 0.01; CV: R = 0.621; *p* < 0.01). There was also a correlation between the BG variability and the total energy daily dose (SD: R = 0.373; *p* = 0.02; CV: R = 0.364; *p* = 0.03). Glycemic variability was higher among patients to whom treatment with adrenalin (*p* = 0.0218) or steroid (*p* = 0.0292) was applied. The BG variability, expressed using SD, was associated with ICU mortality (ROC = 0.806; 95% CI: 0.643–0.917; *p* = 0.0014). The BG variability in the ICU setting arises from the loss of balance between the supplied energy and the applied insulin dose and may be associated with a worse prognosis.

## 1. Introduction

Nondiabetic hyperglycemia is a dangerous metabolic phenomenon in the hospitalized population [1,2,3] and may occur in up to 32–38% of patients treated in the anesthesiology and intensive care unit [2,3]. High glucose levels are the body’s response to stress associated with the underlying disease or surgery. The main processes responsible for stress hyperglycemia are insulin resistance and increased gluconeogenesis [4,5]. This is due to the excessive secretion of hormones such as glucagon, growth hormone, glucocorticoids, catecholamines, and proinflammatory cytokines: interleukin 1, interleukin 6, and TNF-alpha [5,6]. Hyperglycemia causes a number of adverse reactions for the body, such as an increase in free radical expression and associated oxidative damage to cells, as well as a propensity for thrombosis and associated peripheral perfusion abnormalities, abnormal vascular reactivity, or an impaired immune response [5,6]. Hyperglycemia has also been shown to worsen the prognosis of stroke and acute myocardial infarction [7]. The presence of hyperglycemia in the critically ill population is also favored by extrinsic factors such as administered drugs with hyperglycemic effects and patient nutrition [4].

Thus, glycemic control in intensive care becomes a therapeutic aim. A 2001 study by Van Den Berghe et al. demonstrated the advantage of strict over liberal glycemic control [8]. However, in that case, too many hypoglycemic incidents were observed and were associated with an increased risk of death [9]. The 2009 NICE-SUGAR trial provided evidence that more moderate glycemic control is safer and no less effective [10,11]. Hence, the current consensus is that glycemia should be controlled within the range of 140–180 mg/dL [12] and the signal to initiate insulin therapy is its value >200 mg/dL found twice. Moreover, it was noted that inattentive treatment for glycemic disturbances, causing glycemic fluctuations [13] and hypoglycemic episodes [14], is equally dangerous for hospitalized patients. Thus, glycemic variability has become another important parameter with a prognostic value [15]. The aim of this study was to evaluate the variability of glycemia and its underlying determinants as well as to verify the association with the death rate in patients treated in a multiprofile anesthesiology and intensive care unit (ICU).

## 2. Materials and Methods

Medical records of 37 patients hospitalized in a multiprofile ICU between 3 January 2020 and 29 February 2020 were prospectively analyzed. Due to the non-interventional, observational nature of the study, the consent of the Bioethics Committee was not required. The following information was assessed: basic demographic data, reason for admission, admission priority according to the guidelines of the Polish Society of Anesthesiology and Intensive Therapy, patient status on admission according to APACHE II, SAPS II, and SOFA scores, the presence of diabetes mellitus and its type, and treatment before admission to ICU. The following were recorded for the entire treatment period: daily glycemic values, insulin doses used, incidence of hypoglycemia (i.e., <50 mg/dL), energy intake provided by nutrition (intra- and parenteral), as well as energy contained in glucose, propofol, and citrate solutions for renal replacement therapy. The total dose and the mean daily dose were calculated for the above parameters. Blood glucose was measured in all patients on admission and 4 times a day, at 6:00, 12:00, 18:00, and 24:00. If blood glucose values were <200 mg/dL in the first two days of stay, then the measurements were limited and performed only 2 times/day at 6:00 and 18:00. If values >200 mg/dL persisted in 4 consecutive measurements despite insulin therapy, measurements were intensified to 8 measurements at 3:00, 6:00, 9:00, 12:00, 15:00, 18:00, 21:00, and 24:00. Insulin therapy was introduced when the glycemic value in two measurements was ≥200 mg/dL, and the insulin doses were adjusted to keep glycemia control between 140–180 mg/dL. The doses of the most commonly used hypoglycemic drugs in the unit (corticosteroids and epinephrine) were analyzed. The variability of glycemia during the treatment period, defined as (1) the standard deviation (SD) of all measurements taken and (2) the coefficient of variation calculated as the quotient of mean glycemia and SD (×100%), was evaluated. Deaths during the ICU treatment period were analyzed.

Statistical analysis was performed using procedures available in the licensed MedCalc v18.2 software. Quantitative variables were presented as arithmetic mean and standard deviation (normal distribution) or median and interquartile range (IQR, interquartile range) (distribution deviating from normal). The nature of the distribution of quantitative variables was verified with the Shapiro–Wilk test. Qualitative variables were presented as absolute values and percentage. Differences between quantitative variables were assessed using Student’s t-test/analysis of variance or Mann–Whitney U/Kruskal–Wallis test, depending on the number of groups and the character of distribution. For qualitative variables, the chi-square test was used. Correlations were assessed by Pearson’s linear correlation coefficient or Spearman’s rank. The statistical relationship between dichotomous variables was assessed using odds ratio (OR) analysis. Diagnostic accuracy was assessed using ROC curves and area under curve (AUROC). The criterion for statistical significance was *p* < 0.05.

## 3. Results

The study group consisted of 37 patients. The median SAPS II score on admission was 39 (IQR: 28–55). The median treatment duration in the unit was 5 (IQR: 2–8) days. The patients’ characteristics are shown in Table 1. Sepsis was the cause of admission in two (5%) patients. In priority II, according to the Polish Society of Anesthesiology and Intensive Therapy guidelines, three (8%) patients were admitted.

A history of diabetes mellitus was found in nine (24%) patients (all type 2 diabetes mellitus), of whom two (5%) patients were administered insulin and two (5%) patients were receiving oral hypoglycemic drugs. The mean glycemia on admission was 142.1 ± 56.7 mg/dL and was statistically significantly higher in those with a history of diabetes compared to those without diabetes (190.6 ± 82.0 mg/dL vs. 126 ± 34.2 mg/dL; *p* < 0.0001). The mean glycemia during the treatment period was 165.7 ± 40.8 mg/dL and was statistically significantly higher in those with a history of diabetes compared to those without diabetes (213.1 ± 32.6 mg/dL vs. 150.5 ± 30.2 mg/dL; *p* = 0.002). The mean standard deviation (SD) of glycemia during the treatment period was 49.9 ± 21.9 mg/dL and was also statistically significantly higher in subjects with a history of diabetes compared to subjects without diabetes (67.1 ± 15.8 mg/dL vs. 43.1 ± 15.8 mg/dL; *p* = 0.003). The glycemic coefficient of variation (CV) value was 29.4 ± 9.8% and was similar in subjects with and without a history of diabetes (32.0 ± 9.1% vs. 28.5 ± 10.1%; *p* = 0.36).

Insulin was used for glycemic control in 28 (75.7%) subjects. The median total mean insulin dose administered was 97 (5–253) units or 12 (1–36) units/day of hospitalization. Hypoglycemia occurred in six (16.2%) subjects, with a total of 13 cases. Hypoglycemia was over 16 times more common in those who received insulin for glycemic control (OR = 16.5; 95% CI: 1–312; *p* = 0.01).

The median total energy intake administered during the treatment equaled 7820 (IQR: 2126–14274) kcal, with an average of 1078 ± 468 kcal/day of hospitalization. The median total glucose dose administered was 1084 (IQR: 331–1797) g, including an average of 148 ± 73 g glucose/day of hospitalization. Twenty-two (59.5%) patients were treated with glucocorticoids, with a median dose of 850 (550–1344) mg during the entire stay (per hydrocortisone dose). In 15 (40.5%) patients, epinephrine was administered at a median dose of 10.4 (IQR: 1.98–3.7) mg during the entire stay.

Correlations between glycemic variability and selected quantitative variables are shown in Table 2. Statistically significant correlations are observed between BG variability and age, severity of condition at admission according to SAPS II and SOFA (for SD only), and energy, glucose, insulin, steroid, and epinephrine doses.

**Figure 1:**
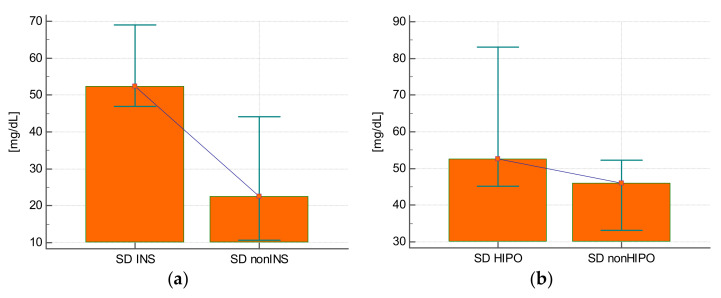

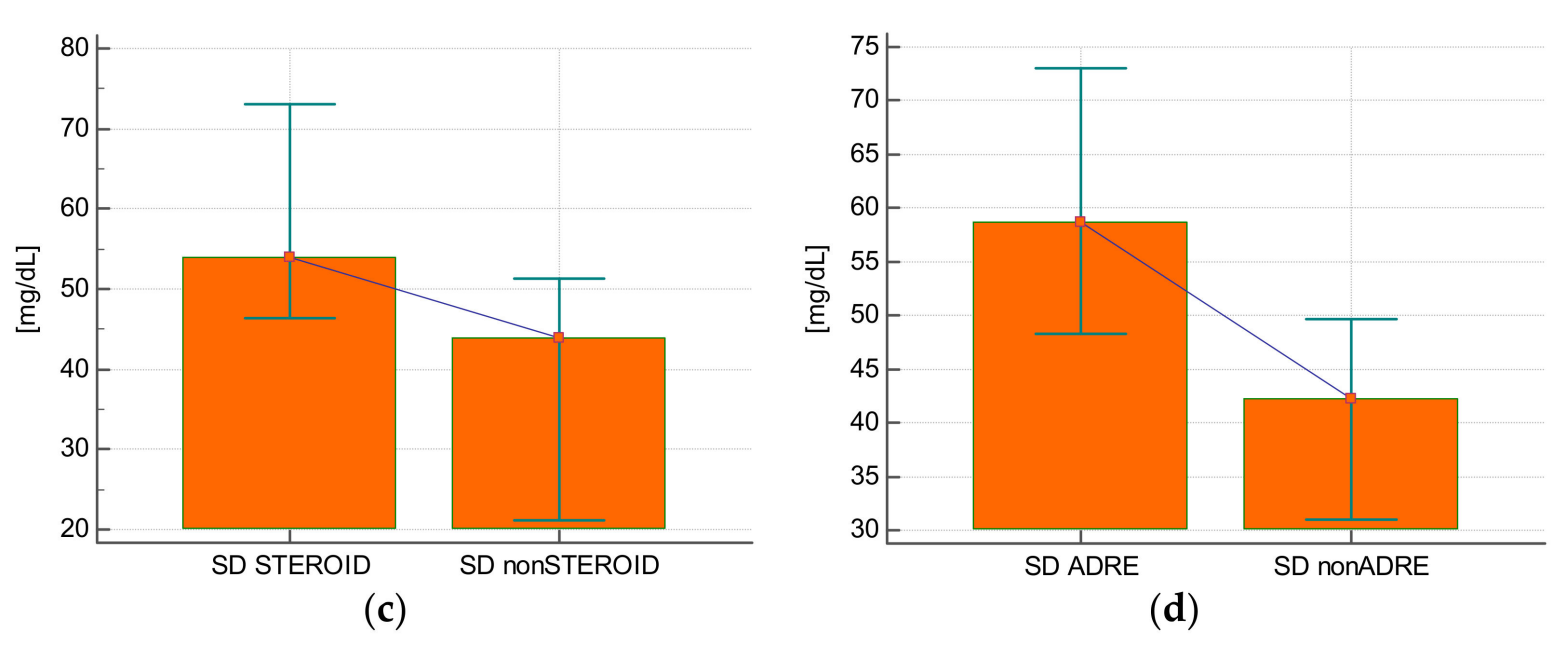
(**a**) The variability of blood glucose defined as the value of standard deviation (SD) in patients receiving insulin during hospitalization (INS) and not requiring insulin therapy (nonINS). (**b**) The variability of blood glucose defined as the value of standard deviation (SD) in patients with hypoglycemia (HIPO) and in patients with no hypoglycemia (nonHIPO) during hospitalization. (**c**) The variability of blood glucose defined as the value of standard deviation (SD) in patients who received glucocorticoids (STEROID) and in those who did not receive glucocorticoids (nonSTEROID). (**d**) The variability of blood glucose defined as the standard deviation (SD) value in patients receiving adrenaline (ADRE) and in patients not receiving adrenaline (nonADRE).

The SD of glycemia during the stay was statistically significantly higher in those who were treated with insulin compared with those who did not require this treatment (25.3 ± 14.3 mg/dL vs. 57.9 ± 17.7 mg/dL; *p* < 0.0001) (Figure 1A), in those who experienced hypoglycemia (63.3 ± 23.1 mg/dL vs. 48.7 ± 20.6 mg/dL; *p* = 0.02) (Figure 1B), and in subjects who received steroids (59.1 ± 20.3 mg/dL vs. 36.6 ± 17.3 mg/dL; *p* = 0.001) (Figure 1C) or epinephrine (61.3 ± 17.2 mg/dL vs. 42.3 ± 21.8 mg/dL; *p* = 0.008) (Figure 1D).

**Figure 2:**
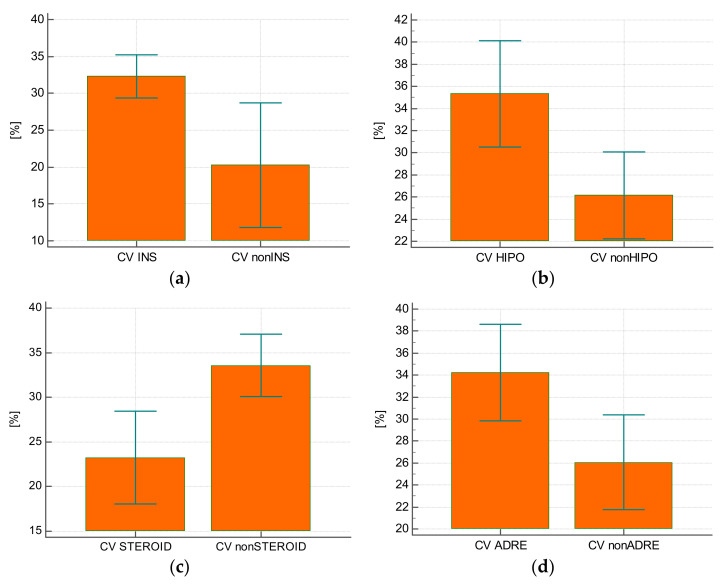
(**a**) Glycemic variability as a value of coefficient of variation (CV) in patients receiving insulin during hospitalization (INS) and in those not requiring insulin therapy (nonINS). (**b**) Glycemic variability as a value of coefficient of variation (CV) in patients with hypoglycemia (HIPO) and in patients with no hypoglycemia (nonHIPO) during hospitalization. (**c**) Glycemic variability as a value of coefficient of variation (CV) in patients who received glucocorticoids (STEROID) and in those who did not receive glucocorticoids (nonSTEROID). (**d**) Glycemic variability as a value of coefficient of variation (CV) in patients receiving adrenaline (ADRE) and in those not receiving adrenaline (nonADRE).

The CV value of glycemia was statistically significantly higher in subjects who received insulin compared with those who did not require this treatment (32.3 ± 7.5% vs. 20.3 ± 11%; *p* = 0.0007) (Figure 2A), in subjects who experienced hypoglycemia (38.8 ± 8.9% vs. 27.6 ± 9%; *p* = 0.005) (Figure 2B), and in those who received steroids (33.6 ± 7.8% vs. 23.2 ± 9.4%; *p* = 0.0009) (Figure 2C) or epinephrine (34.2 ± 7.9% vs. 26.1 ± 9.8%; *p* = 0.01) (Figure 2D).

**Figure 3:**
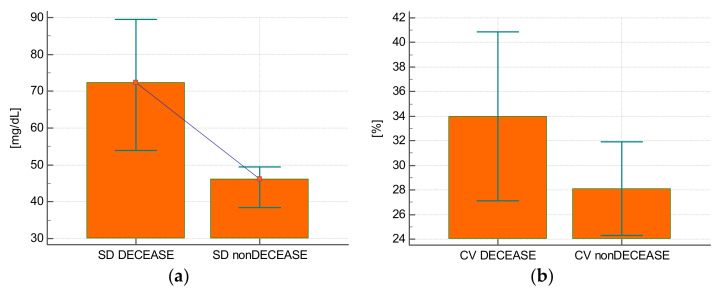
(**a**) The variability of blood glucose defined as the value of standard deviation (SD) in patients who died (DECEASE) and patients who survived (nonDECEASE). (**b**) Glycemic variability as a value of coefficient of variation (CV) in patients who died (DECEASE) and patients who survived (nonDECEASE).

Eight (21.6%) patients died during their stay in the intensive care unit. The glycemia SD value was higher in those who died (68.9 ± 18.6 mg/dL vs. 44.7 ± 20 mg/dL; *p* = 0.004) (Figure 3A). The SD allowed to predict the risk of death with a good diagnostic accuracy (AUROC = 0.806; 95% CI: 0.643–0.917; *p* = 0.0014). The glycemia CV was comparable for those who died and those who were discharged from the unit (34 ± 8.2% vs. 28.1 ± 10%; *p* = 0.13) (Figure 3B). The CV was not predictive of the risk of death (AUROC = 0.690; 95% CI: 0.517–0.831; *p* = 0.09).

## 4. Discussion

The aim of this study was to evaluate the variability of glycemia, its underlying determinants, and to verify its association with the incidence of death in patients treated in the anesthesiology and intensive care unit.

The analyses showed that both mean glycemia on admission and mean glycemia during the stay were significantly higher in patients with a history of diabetes. The mean SD of glycemia during the stay was significantly higher in patients with a history of diabetes compared with patients without this illness, while the CV of glycemia was similar. This stays in agreement with previous studies showing that HBA1c levels are strongly associated with mean glycemic value on admission to the ICU, increase in glycemic variability, and incidence of hypoglycemia [16]. The authors of the cited studies highlight individuals with prediabetic conditions, who have not yet developed a tolerance for acute hyperglycemic states, and therefore identify this group as requiring greater attention in achieving optimal glycemic control.

Hypoglycemia was sixteen times more frequent in patients receiving insulin therapy. This is a dangerous prognostic phenomenon for patients [17] because even mild hypoglycemia (without signs of neuroglycopenia) increases mortality. [18] Glucose control monitoring (GCM), which significantly reduces hypoglycemic incidents, seems to be a solution to this problem [19]. Improvements in glycemic variability when GCM is used have also been documented in patients diagnosed with diabetes [20]. However, GCM has been shown to not always reduce glycemic variability. It does not affect variability as long as there is an implemented and well-adhered-to insulin-dosing algorithm in the unit, combined with an experienced unit team and frequent blood glucose measurements. However, it is useful in less experienced or frequently rotating teams [21]. We also showed a positive correlation between SD of glycemia and the severity of condition on admission (according to SAPS II and SOFA) and patient age. Previous authors have reached similar conclusions, in groups of patients with and without a prior diagnosis of diabetes [22]. Higher mean glucose levels in patients with SOFA ≥9 points are correlated with stress-induced hyperglycemia, and thus increased gluconeogenesis and insulin resistance, as well as with older patient age, which also translates into increased insulin resistance compared to younger groups [22,23,24].

The SD and CV values of glycemia were higher if the patient developed a hypoglycemic episode, and was treated with insulin, steroids, or epinephrine. This is in agreement with the observation that a bolus of hydrocortisone is associated with greater variability in glycemia, as well as greater variability in insulin levels and greater need for boluses of this drug [25]. CV has also been identified in previous studies as an independent predictor better associated with hypoglycemia than SD is [26].

The relationship between glycemic variability and total energy intake throughout the day was also found to be statistically significant. This is supported by previous studies that showed a negative correlation between total caloric supply and total serum insulin concentration, and a positive correlation between caloric supply and exogenously administered insulin [27]. Parenteral nutrition, more often than enteral nutrition, induces hyperglycemia. This may be due to the lack of incretin effect, as well as a more pronounced proinflammatory response [28].

Finally, it was the SD value of glycemia that proved to be a better predictor of death in our study population. The SD value may also be a better indicator of variability than CV is when we aim to assess the potential effects of glycemic variability without the influence of hypoglycemia [29].

### Limitations of Inference

Our study has several limitations. First of all, it is a single-center study on a heterogeneous, relatively small number of participants. However, similar works in this context exist in the literature, and our observations are consistent with the results of other researchers. Secondly, insulin resistance was not assessed, yet it may be of unmitigated importance for the pathophysiological interpretation of the results. The degree of diabetes control by HBA1c was also not assessed; however, the presence of diabetes had no significant effect on glycemic variability. This is due to the different background of hyperglycemia in chronically present diabetes from that in the acute, critical condition. The lack of an automated glycemic delivery system (SGC, Space Glucose Control^®^ system) can be considered as another limitation of work. However, SGC cannot fully eliminate the risk of glycemic fluctuations, as it requires frequent glycemic measurements and their entry into the system. Lastly, blood glucose measurements were performed both in arterial blood and in capillary blood drawn from a finger, which carries the risk of measurement error, with a margin of measurement error of up to 20%.

## 5. Conclusions

High glycemic variability results from an imbalance between energy delivery and insulin therapy and may be associated with poor prognosis of patients treated in the anesthesiology and intensive care unit.

## Figures and Tables

**Table 1:** Characteristics of patients.

Variable	Value
Men/Female	22 (59.5%)/15 (40.5%)
Age (years)	62 ± 15
Weight (kg)	80 ± 15
Body Mass Index (kg/m^2^)	27.5 ± 5.2
Height (cm)	170 (160–180)
APACHE II on admission (points)	19.5 (9–23.5)
SAPS II on admission (points)	39 (27–55)
SOFA on admission (points)	10 (8–12)
The cause of admission:	
Medical	23 (62%)
Surgical	14 (38%)

**Table 2:** The correlation between blood glucose variability and selected quantitative variables.

Variable	Glucose Variability— Standard Deviation (mg/dL)	Glucose Variability— Coefficient of Variation (%)
Patients age (years)	R = 0.520 *p* = 0.001	R = 0.417 *p* = 0.01
Body mass index (kg/m^2^)	R = 0.254 *p* = 0.17	R = 0.153 *p* = 0.42
APACHE II on admission (points)	R = 0.217 *p* = 0.31	R = 0.264 *p* = 0.21
SAPS II on admission (points)	R = 0.519 *p* = 0.009	R = 0.333 *p* = 0.11
SOFA on admission (points)	R = 0.568 *p* = 0.004	R = 0.374 *p* = 0.07
Glycemia on admission (mg/dL)	R = 0.204 *p* = 0.23	R = −0.161 *p* = 0.35
Total energy dose/day (kcal)	R = 0.373 *p* = 0.02	R = 0.364 *p* = 0.03
Total glucose dose/day (g)	R = 0.195 *p* = 0.25	R = 0.363 *p* = 0.03
Total insulin dose/day (j)	R = 0.559 *p* = 0.0003	R = 0.621 *p* < 0.0001
Total steroid dose/day (mg)	R = 0.551 *p* = 0.0078	R = 0.465 *p* = 0.0292
Total dose of adrenaline/day (mg)	R = 0.118 *p* = 0.6757	R = 0.586 *p* = 0.0218

## Data Availability

The data presented in this study are available on request from the corresponding author.
